# Is low total cholesterol levels associated with suicide attempt in depressive patients?

**DOI:** 10.1186/s12991-017-0144-4

**Published:** 2017-04-17

**Authors:** A. Messaoud, R. Mensi, A. Mrad, A. Mhalla, I. Azizi, B. Amemou, I. Trabelsi, M. H. Grissa, N. Haj Salem, A. Chadly, W. Douki, M. F. Najjar, L. Gaha

**Affiliations:** 1 Research Laboratory ‘Vulnerability to psychotic disorders LR 05 ES 10’, Department of Psychiatry, Monastir University Hospital, University of Monastir, Monastir, Tunisia; 2 Laboratory of Biochemistry-Toxicology, Monastir University Hospital, University of Monastir, Monastir, Tunisia; 3 Department of Emergency, Monastir University Hospital, University of Monastir, Monastir, Tunisia; 4 Department of Forensic Medicine, Monastir University Hospital, University of Monastir, Monastir, Tunisia

**Keywords:** Depression, Suicide, Cholesterol, Biological marker

## Abstract

**Background:**

Patients with major depressive disorder (MDD) have a high risk of suicide. Many pathophysiological factors involved in MDD and suicide such us a low cholesterol levels have been associated with MDD and increased vulnerability to suicide. In this study, we investigate the relation between lipid parameters and suicide risk in patients with MDD.

**Methods:**

Plasma levels of total cholesterol, triglycerides, and high-density lipoprotein cholesterol (HDL-c) and low-density lipoprotein cholesterol (LDL-c) were determined in 160 patients meeting the DSM-IV-TR criteria for MDD (110 patients without suicidal behavior and 52 suicidal attempters) and 151 healthy controls.

**Results:**

A significant decrease in plasma cholesterol levels was observed in the group of suicidal depressive patients compared to those without suicidal behavior (*p* < 0.001). For the other lipid levels (triglycerides, HDL cholesterol, and LDL cholesterol), there were no significant differences between suicidal and non-suicidal patients.

**Conclusions:**

Our study showed a significant decrease in plasma cholesterol levels in suicidal patients. This result support the hypothesis of the association of low plasma cholesterol level and suicidal behavior in patients with major depressive disorder.

## Background

Major depressive disorder is a common, recurrent, and chronic psychiatric illness. Several factors seem to be implicated in the pathophysiology of depression, and a contribution of genetic, environmental, and social factors is greatly discussed [1]. In a report on the Tunisian Health System [2], the Tunisian Ministry of the Public Health estimated that more than 8.2% of the population suffers from depression.

Severity of depression is accompanied by a high suicidal risk. Indeed, studies using the technique of psychological autopsy found that more than 90% of suicidal patients are affected by one or more psychiatric disorders, such as major depressive disorder (MDD), at the time of the suicidal act [3]. In this same context, Roy [4] found that 45–75% of suicide victims suffered from depression at the time of their death.

In Tunisia, especially after the revolution of January 14th, 2011, suicide rates continue to increase. In 2015, FTDES [5] recorded 549 cases of suicide and suicide attempts with an increase of 170.4% compared to 2014. This is an alarming and worrying phenomenon requiring an improvement in the strategies of prevention of this tragedy.

Many researchers have been interested in identifying biological markers that could be associated with depressive disorder and suicidal behavior, and could be used as an additional tool for prevention actions [6–8].

Several studies have suggested that an alteration in the lipid profile can occur to people with these kinds of disorders [9–13]. Some researches have concentrated on the relationship between plasma or serum cholesterol levels and suicide; however, conflicting results have been reported [14–16]. Some of these studies have found an association between low cholesterol levels and increased risk of suicide [17–19], and others have reported negative association of cholesterol with suicidal ideation [20, 21], current parasuicide [15, 22], history of attempted suicide [23], and completed suicide [24].

Besides total cholesterol, other researches have investigated the link between triglycerides, HDL cholesterol, LDL cholesterol, depression, and suicidal behavior [25–27]. Some studies have documented that lower triglyceride levels were significantly associated with suicidal tendency in patients with depression [25, 26]. However, for HDL cholesterol or LDL cholesterol, Cantarelli et al. did not find any difference between depressed patients with or without suicidal behaviors [26].

In the present study, we examined the lipid and lipoprotein profiles in Tunisian adults with major depressive disorder and with or without suicide attempt. We aimed to verify whether an alteration in lipid profile increases the risk of suicide in patients with major depressive disorder or not.

## Methods

### Subjects

This study has included 162 patients with major depressive disorder according to DSM-IV and 151 controls. Patients were classified into two groups: 110 patients with a major depressive episode (MDD) without suicidal behavior recruited during the consultations in the Department of Psychiatry of the University Hospital of Monastir; and 52 MDD suicide attempters recruited when admitted to the emergency department after a suicide attempt. Data were collected using a data sheet containing socio-demographic, clinical, and therapeutic information of the patient. Only patients between 20 and 60 years of age were involved in this study. We excluded from this study patients treated for dyslipidemia, hypertension, or diabetes. The majority of patients received antidepressant treatment according to their individual clinical needs.

The control group consisted of 151 volunteer subjects without psychiatric or endocrinological diagnoses matched for age, gender, BMI, tobacco, and alcohol addiction. Those with a history of suicidal act or major medical illness were excluded from this group. General characteristics of the subjects are shown in Table 1. Our study was approved by the Ethical Committee of the University Hospital of Monastir.

**Table 1:** General characteristics of the study population

Characteristics	Non-suicidal MDD patients( *N* = 110)	MDD suicide attempters (*N *= 52)	Normal controls (*N* = 151)	*p*
Gender (men/women)	35/75	19/33	50/101	0.837
Age (years)	44.33 ± 10.50	29.84 ± 8.78	38.92 ± 13.28	0.000
BMI	26.24 ± 2.57	25.65 ± 3.61	25.58 ± 4.10	0.440
Addiction				
Cigarette smoking				
Smokers	20 (19.19%)	14 (38.9%)	24 (15.9%)	0.034
Non-smokers	90 (81.81%)	36 (61.1%)	127 (84.1%)	
Alcoholic beverages				
Consumers	12 (10.9%)	11 (21.6%)	16 (10.5%)	0.079
Non-consumers	98 (89.1%)	40 (78.4%)	135 (89.5%)	
Diagnosis (DSM-IV)				
Major depressive disorder, single episode	31(28.2%)	30 (57.7%)	–	–
Major depressive disorder, recurrent	97 (71.8%)	22 (42.3%)	–	–
Treatment				
ISRS	47 (42.7%)	18 (34.6%)	–	–
Tricyclics	42 (38.2%)	12 (23.1%)	–	
Mood stabilizers	31 (28.2%)	15 (28.9%)	–	
Antipsychotics	17 (15.5%)	5 (9.6%)	–	
Benzodiazepine	19 (17.3%)	8 (15.4%)		

**Fig. 1:**
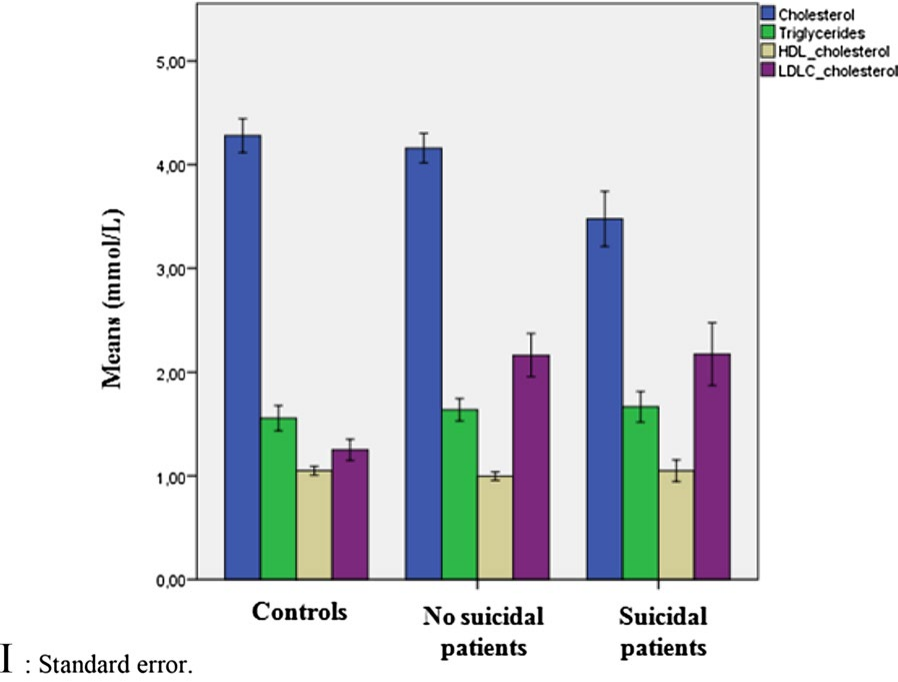
Plasma lipids levels in patients with and without suicide attempts and controls

Plasma levels of total cholesterol, triglycerides, HDL-c, and LDL-c are presented in Fig. 1

### Psychiatric assessment

The diagnosis of the MDD was made using the Diagnostic and statistical manual of mental disorders (DSM-IV). We have also used other scales such as Hamilton Depression Rating Scale (HDRS) and the Beck’s Suicidal Ideation Scale (SSI) to rate the severity of depressive symptoms.

### Biochemical measurements

Blood samples were drawn after overnight fasting. For suicidal patients, samples were collected within 24 h after the suicide attempt. Plasma levels of total cholesterol (TC), triglycerides (TG), and high-density lipoprotein cholesterol (HDL-c) were determined by enzymatic methods, and low-density lipoprotein cholesterol (LDL-c) was calculated by the Friedewald equation.

### Statistical analysis

The statistical analyses were performed using SPSS 21.0 for Windows. All variables were presented as mean ± standard deviation (SD). Categorical variables were presented as the raw number and percentage (%). To compare total cholesterol, LDL-c, HDL-c. and triglycerides, we used the analysis of covariance (ANCOVA) and the student *t* test. ROC analysis for biological variables was used to find cutoff points, sensitivity, specificity, and positive and negative predictive values. Odds ratios were calculated. The Pearson’s and Spearman’s correlation coefficients were calculated to evaluate the correlations between biological and clinical (HDRS and SSI) variables. Differences were considered as significant if the *p* value was <0.05.

## Results

Demographic characteristics of patients and controls groups are presented in Table 1. Suicidal patients with major depressive disorder were significantly younger than patients without suicidal behavior (*p* < 0.001). Most of the suicide attempters were women (*p* ≤ 0.05). The majorities of our participants were not smokers and do not consume alcohol. There was no significant difference between patients regarding BMI (Table 1).

The mean plasma level of cholesterol was significantly lower among the suicide attempters than the depressive group (3.47 ± 0.95 vs 4.15 ± 0.75 mmol/L) and the control group (4.27 ± 1.01 mmol/L). The difference between the MDD suicidal patients group, non-suicidal patients, and control groups was significant (*p* < 0.001), but not between non-suicidal patients and control group (*p* = 0.211). The increase in the mean plasma values of triglycerides in all depressed patients, regardless to suicidal behaviors, compared to controls was statistically not significant (*p* > 0.05) (Fig. 1). However, the increase in the mean plasma concentration of LDL-c in depressive group, attempters and non-attempters, was statistically significant when compared with controls subjects (*p* < 0.05). For the HDL cholesterol level, we observed a slight decrease in all patients with major depressive disorder which is not significant when compared to the control group (*p* < 0.05). No significant difference, after adjusting for age and cigarette smoking, in all lipids parameters was noticed between the suicide attempts and completions groups.

**Fig. 2:**
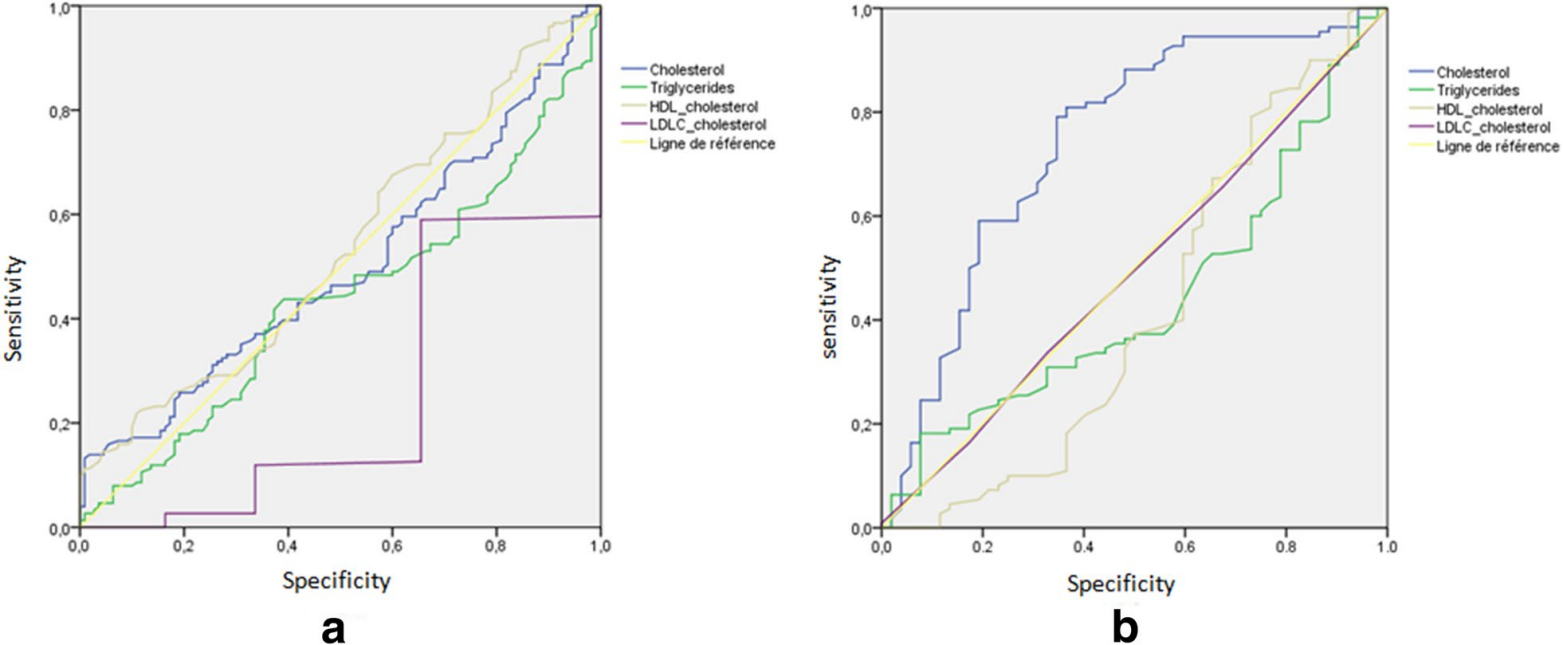
Receiver operating characteristic curves (ROC) constructed using the lipid parameters. **a** Non suicidal depressive patients. **b** suicide attempters

The analysis of ROC curves of these four lipid parameters in the non-suicidal patients (a) showed that the cholesterol curve was near the diagonal with an area under the curve on the order of 0.511. The significant difference in LDL cholesterol level between depressive and control group did not appear in the ROC curve. However, in the suicidal patients (b), the curve of cholesterol was the most discriminant. The area under the curve of cholesterol was the highest compared with the other lipid parameters (Fig. 2).

For total cholesterol, a threshold value of 3.47 mmol/L was chosen for the subsequent analyses. The frequencies of distribution of lipid parameters showed that more than the half of MDD suicidal patients (55.7%) had a mean level of total cholesterol below 3.47 mmol/L. For these patients, the risk to commit a suicide attempt is increased five times with a plasma total cholesterol level less than 3.47 mmol/L. Similarly for HDL-c, patients with plasma level lower then 1.17 mmol/L have a suicidal risk multiplied by two than those with a level higher than this threshold value. Regarding triglycerides and LDL-c, no significant difference in the frequency of disturbance was observed between the patients with and without suicidal behaviors (Table 2).

**Table 2:** Association between disturbances in lipid parameters and the suicidal behavior in the study population

Parameters	Patients without suicide attempt		Patients with suicide attempt		*p*	Odds ratio	IC 95%
	*N*	%	*N*	%			
Total cholesterol (mmol/L)							
<3.47	19	17.3	29	55.7	0.000	5.039	2.889–12.620
≥3.47	91	82.7	23	54.3			
Triglycerides (mmol/L)							
<1.70	72	65.4	29	55.7	0.155	0.665	0.579–1.305
≥1.70	38	34.6	23	44.3			
HDL-c (mmol/L)							
<1.17	26	23.6	23	54.3	0.007	2.562	1.270–5.171
≥1.17	84	76.4	29	55.7			
LDL-c (mmol/L)							
<2.50	37	33.6	17	32.7	0.527	0.958	0.475–1.933
≥2.50	73	66.4	35	67.3			

When patients were classified according to gender, BMI, tobacco, smoking, and alcohol addiction, there were no statistically significant differences between the plasma triglycerides, HDL cholesterol, and LDL cholesterol in suicidal and non-suicidal MDD patients. Yet, the mean plasma cholesterol concentration was significantly reduced in suicidal women when compared with corresponding value in MDD non-suicidal women (Table 3).

**Table 3:** Plasma lipid levels according to demographic, clinical, and therapeutic data of MDD patients with or without suicide attempt

Parameters	Total cholesterol (mmol/L)	Triglycerides (mmol/L)	HDL-c (mmol/L)	LDL-c (mmol/L)
Gender				
Men				
Patients without suicide attempt	4.0 ± 0.79	1.67 ± 0.60	1.01 ± 0.23	2.17 ± 1.07
Patients with suicide attempt	3.59 ± 0.92	1.65 ± 0.41	1.10 ± 0.28	2.36 ± 1.01
Women				
Patients without suicide attempt	4.22 ± 0.73	1.62 ± 0.59	0.98 ± 0.21	2.16 ± 1.11
Patients with suicide attempt	3.40 ± 0.97*	1.67 ± 0.59	1.01 ± 0.41	2.06 ± 1.11
BMI (kg/m^2^)				
<25				
Patients without suicide attempt	4.12 ± 0.65	1.63 ± 0.60	0.99 ± 0.20	2.00 ± 1.07
Patients with suicide attempt	3.51 ± 0.68	1.66 ± 0.56	1.09 ± 0.58	2.32 ± 1.10
[25–30]				
Patients without suicide attempt	4.13 ± 0.76	1.65 ± 0.60	0.99 ± 0.22	2.29 ± 1.09
Patients with suicide attempt	3.46 ± 1.10	1.68 ± 0.59	1.04 ± 0.42	2.17 ± 1.08
≥30				
Patients without suicide attempt	4.04 ± 1.24	1.59 ± 0.56	1.07 ± 0.162	1.71 ± 1.11
Patients with suicide attempt	3.80 ± 0.99	1.58 ± 0.24	0.93 ± 0.32	1.80 ± 1.09
Tabac				
Non-smokers				
Patients without suicide attempt	4.19 ± 0.76	1.62 ± 0.58	0.99 ± 0.20	2.12 ± 1.11
Patients with suicide attempt	3.48 ± 0.96*	1.69 ± 0.59	1.08 ± 0.38	2.02 ± 1.05
Smokers				
Patients without suicide attempt	4.10 ± 0.77	1.77 ± 0.65	0.98 ± 0.26	2.35 ± 1.08
Patients with suicide attempt	3.45 ± 0.91*	1.60 ± 0.36	0.97 ± 0.34	2.50 ± 1.16
Alcohol				
Non-consumers				
Patients without suicide attempt	4.19 ± 0.74	1.64 ± 0.60	0.99 ± 0.21	2.18 ± 1.13
Patients with suicide attempt	3.60 ± 0.89*	1.66 ± 0.58	1.04 ± 0.34	2.12 ± 1.09
Consumers				
Patients without suicide attempt	3.84 ± 0.80	1.60 ± 0.50	1.00 ± 0.24	2.00 ± 0.73
Patients with suicide attempt	3.03 ± 1.13*	1.67 ± 0.28	1.04 ± 0.48	2.36 ± 1.12
Clinical subtypes				
Patients without suicide attempt				
Single episode	4.02 ± 0.74	1.64 ± 0.76	0.99 ± 0.21	2.20 ± 1.04
Recurrent episode	4.20 ± 0.75	1.64 ± 0.61	0.99 ± 0.22	2.14 ± 1.11
Patients with suicide attempt				
Single episode	3.47 ± 0.98	1.62 ± 0.35	1.03 ± 0.41	2.06 ± 1.04
Recurrent episode	3.47 ± 0.94	1.75 ± 0.62	1.07 ± 0.32	2.31 ± 1.12
Severity of MDD				
Patients without suicide attempt				
Mild	4.10 ± 0.73	1.69 ± 0.69	1.00 ± 0.21	2.16 ± 1.08
Moderate	4.18 ± 0.76	1.59 ± 0.59	0.99 ± 0.22	2.20 ± 1.12
Severe	4.27 ± 0.90	1.83 ± 1.22	0.93 ± 0.26	1.50 ± 0.57
Patients with suicide attempt				
Mild	–	–	–	–
Moderate	3.51 ± 0.71	1.62 ± 0.86	0.98 ± 0.63	2.08± 0.98
Severe	3.45 ± 0.98	1.69 ± 0.94	1.11± 0.47	2.24± 1.10
Treatments				
Patients without suicide attempt				
ISRS	4.06 ± 0.84	1.58 ± 0.55	0.98 ± 0.19	2.21 ± 1.06
Tricyclics	4.28 ± 0.70	1.66 ± 0.68	0.99 ± 0.23	2.23 ± 1.20
Others	4.09 ± 0.46	2.17 ± 1.06	0.98 ± 0.22	2.00 ± 0.81
Patients with suicide attempt				
ISRS	3.49 ± 0.21	1.57 ± 0.62	1.05 ± 0.27	2.15 ± 1.13
Tricyclics	3.64 ± 0.75	1.94 ± 0.83	0.95 ± 0.64	2.52 ± 0.67
Others	3.25 ± 0.06	1.65 ± 0.33	1.29 ± 0.14	1.50 ± 0.70

* *p* < 0.05

With regard to episodes of depression, the patients were classified into two categories: single and recurrent episode(s). We did not find significant differences in lipid concentrations among suicidal and non-suicidal MDD patients. As far as concerned about the severity of depression, there were no significant changes in the plasma lipid levels between mild, moderate, and severe depression in all depressed patients. Also there were no significant differences of the plasma lipid levels in relation to the treatment (Table 3).

As shown in Table 4, plasma lipid and lipoprotein levels did not correlate with Hamilton Depression scores (HDRS) and the Beck’s suicidal ideation scale (SSI) in non-suicidal patients. However, negative correlation between plasma levels of total cholesterol and the suicidal ideation (SSI) was found in suicidal patients.

**Table 4:** Correlation between lipids levels and clinical scales scores (HDRS and SSI)

	CT	TG	HDL-c	LDL-c
MDD no suicidal patients				
HDRS				
−*r*	0.119	0.067	0.064	−0.045
−*p*	0.215	0.485	0.508	0.637
SSI				
−*r*	0.008	0.077	−0.023	−0.071
−*p*	0.936	0.423	0.808	0.462
MDD suicidal patients				
HDRS				
−*r*	0.101	−0.040	0.215	0.059
−*p*	0.563	0.817	0.215	0.737
SSI				
−*r*	−0.355*	−0.149	0.160	0.215
−*p*	0.037	0.394	0.359	0.215

*r* Spearmen coefficient correlation

* p < 0.05

## Discussion

According to our results, total plasma cholesterol levels among suicidal depressive patients were significantly lower than those among control depressive patients or control subjects. The low plasma total cholesterol level was significantly related to suicidal behavior. We did not find a difference in the level of plasma cholesterol between the suicide attempters and suicide death groups. This finding agrees with those of previous studies [28–30] but not with those of Baek et al. [31] and Bartoli et al. [32]. The mechanisms of action adduced by these studies show the direct relationship between low cholesterol levels and poor serotonin uptake and the decrease in brain-cell-membrane viscosity. So it seems possible that cholesterol can be used clinically to predict suicide risk in patients with MDD. We employed ROC analysis to test plasma total cholesterol as a discriminate parameter between suicidal and non-suicidal patients. Since the area under the curve is 0.742, cholesterol plasma level can discriminate between these two groups. According to the curve, the plasma total cholesterol level of 3.47 mmol/L is a good cutoff for possible risk of suicide for patients with MDD. So for patients with depression, a plasma cholesterol level less than 3.47 mmol/L indicates a possible risk of suicide and we must take preventive measures in order to anticipate the passage to the suicidal act.

Our data showed also that the significant difference in the level of cholesterol between suicidal and non-suicidal depressive patients existed with regard to the demographic characteristics, witch means that these characteristics did not interfere in this alteration of plasma cholesterol level in suicidal patients. Cantarelli et al. [26], for example, did not also find correlation between BMI and lipid parameters of mood disorder subjects with and without history of suicide. It has been reported also by some studies, related to the gender, that male gender is associated with lower cholesterol levels in various psychiatric disorders [33] and this can be explained by the fact that substance abuse, including alcoholism, may be associated with altered cholesterol serum levels [34]. We reported also that although smokers suffer from depression more than non-smokers and that smokers are twice more likely to commit suicide than non-smokers, we noticed the same difference in lipid profile between suicidal and non-suicidal patients regardless of their smoking addiction.

Our study noticed also that there was no significant changes in triglycerides and HDL-c levels between the depressed patients with and without suicide behavior and the control group. These data are in agreement with some other studies in the literature [35–37] but not with others who found a significant decrease in serum triglycerides in suicide attempters more than non-attempters [26]. In their study, Cantarelli et al. [26] found that serum triglycerides and leptin may act as a suicidal marker in patients with mood disorders. For HDL-c, a significant association between low HDL-c and increased prevalence of suicide attempts was observed in women [38].

The increase of LDL cholesterol level observed in all depressive patients, with or without suicide attempt, is in agreement with some studies [39]. However, Huang [40] did not find an association between plasma LDL-c and depression or suicidality. In our study, this increase in LDL-c plasma level was associated with depressive illness but not suicidal behavior. We noted that Fischer et al. [41] had found an association between higher LDL-c levels and the long allele coding for serotonin transporter gene in women.

With regard to demographic parameters taken into consideration in our study, the result showed that the risk of suicide attempt is higher in females. Our sample included more females than males with a history of attempted suicide. These data were confirmed by Onuegbu et al. [42]. It is important to mention that the total plasma cholesterol level measured in women after a suicide attempt is significantly lower than women who had not attempted suicide. This result was found also by Guillem et al. [22].

Regarding the way used to commit suicide, in our study, drug poisoning and/or raticide were the most common modes used by suicide attempters (83.3%). On the other hand, the majority of suicide deaths commit suicide by hanging (50%) or by immolation (30%). These results agree with those of Ghachem et al. [3] where suicide deaths use more decisive, fatal, and violent methods.

We did not find any difference in lipid concentrations depending on the kind of antidepressants used. Vuksan–Cusa et al. [25] have reported that medication is not the only contributor to changes in lipid profile.

No correlation was found in non-suicidal patients between lipid plasma levels and clinical assessments of depression and suicidal behavior. This result is in agreement with data of Onuegbu et al. [42]. In suicidal patients, we found a negative correlation between total cholesterol levels and SSI score, meaning that suicidal ideations are present more in patients with low total cholesterol levels.

Some methodological limitations should be considered in the interpretation of our results: the small sample size of the groups studied, especially the suicidal deaths, which lead to a limitation on the amount of independent variables we could use. Also the significant difference in age between suicidal and no suicidal patients can also confound the results.

## Conclusion

In conclusion, our study’s result adds to a majority of research showing the association between suicidal behavior and lower total plasma cholesterol levels. So we support the role of plasma cholesterol as a biological state marker for the assessment of suicide risk in patients with a major depressive episode. We recommend to check if an increase of the cholesterol supplement in the diet can reduce the suicide risk in depressive patients.

## Abbreviations

MDD: major depressive disorder
TC: total cholesterol
TG: triglycerides
HDL-c: high-density lipoprotein cholesterol
LDL-c: low-density lipoprotein cholesterol
BMI: body mass index
HDRS: Hamilton depression rating scale
SSI: scale of suicidal ideation
